# Supplementary material for: Low circulating miR-5193 promotes PD-L1-mediated immune evasion and relates to immune checkpoint inhibitor response in esophageal cancer
Source: Mol Ther Nucleic Acids. 2026 Aug 12;37(3):103059. doi: 10.1016/j.omtn.2026.103059 (PMC13542498; doi:10.1016/j.omtn.2026.103059)
Supplement: Supplementary file 1 [file mmc1.pdf]

OMTN, Volume *37*

Supplemental information

Low circulating miR-5193 promotes PD-L1-mediated immune evasion and relates to immune checkpoint inhibitor response in esophageal cancer

Ryo Ishida, Shuhei Komatsu, Hajime Kamiya, Takuma Ohashi, Taisuke Imamura, Jun Kiuchi, Keiji Nishibeppu, Yusuke Takashima, Hiroshi Arakawa, Masateru Yamauchi, Satoshi Hamada, Hiroyuki Kanazawa, Hiroki Shimizu, Tomohiro Arita, Toshiyuki Kosuga, Hirotaka Konishi, Hitoshi Fujiwara, Tomoko Iehara, Hitoshi Tsuda, and Atsushi Shiozaki

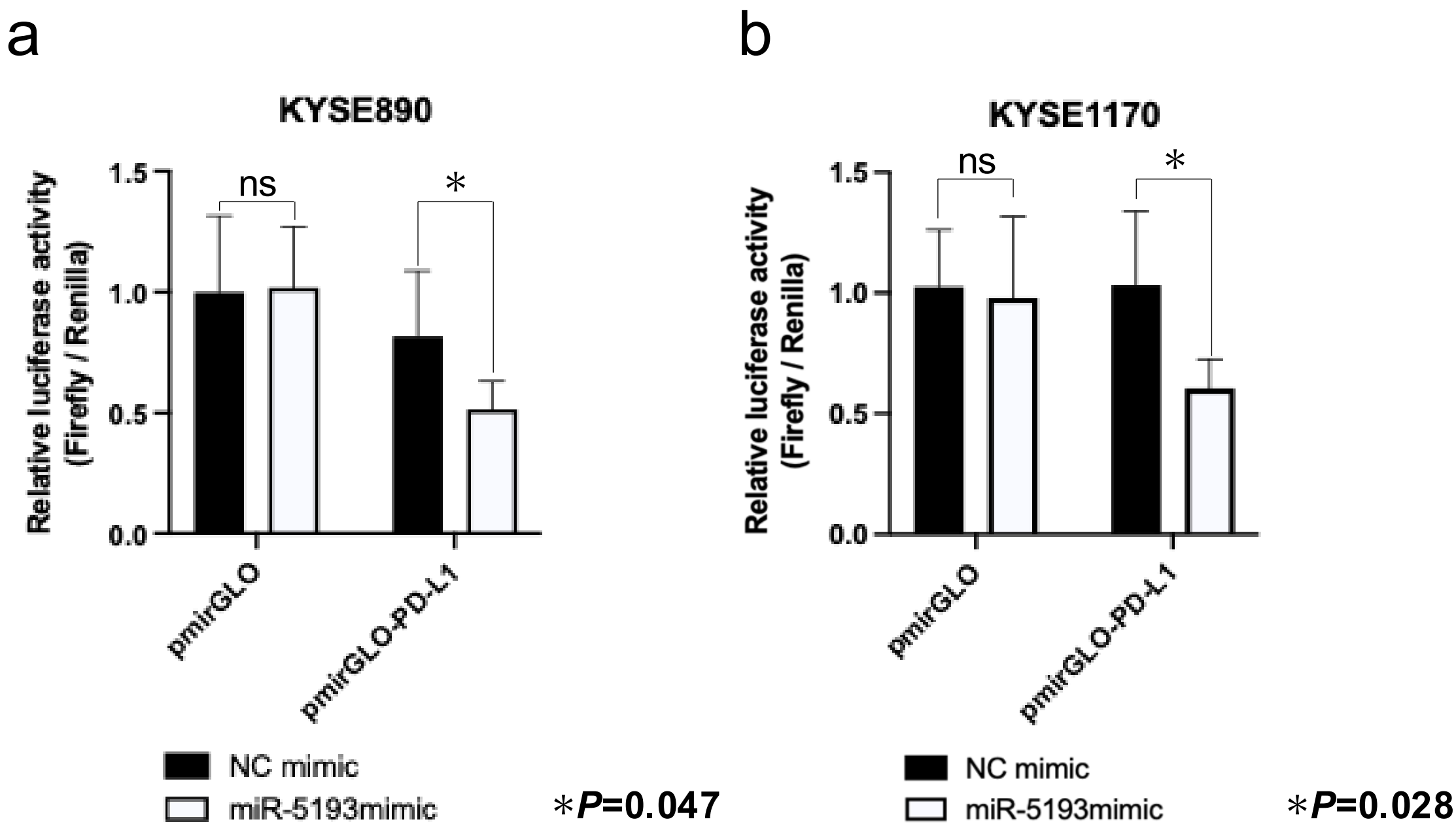

Figure S1

KYSE890 and KYSE1170 cells were co-transfected with an empty pmirGLO vector or a pmirGLO vector containing the PD-L1/CD274 3′ untranslated region (3′UTR), together with miR-5193 mimic or negative-control mimic. Luciferase activity was measured 48 h after transfection. Firefly luciferase activity was normalized to Renilla luciferase activity, and the relative luciferase activity of the negative-control mimic was set to 1 for each reporter construct. miR-5193 significantly reduced the luciferase activity of the PD-L1/CD274 3′UTR reporter.

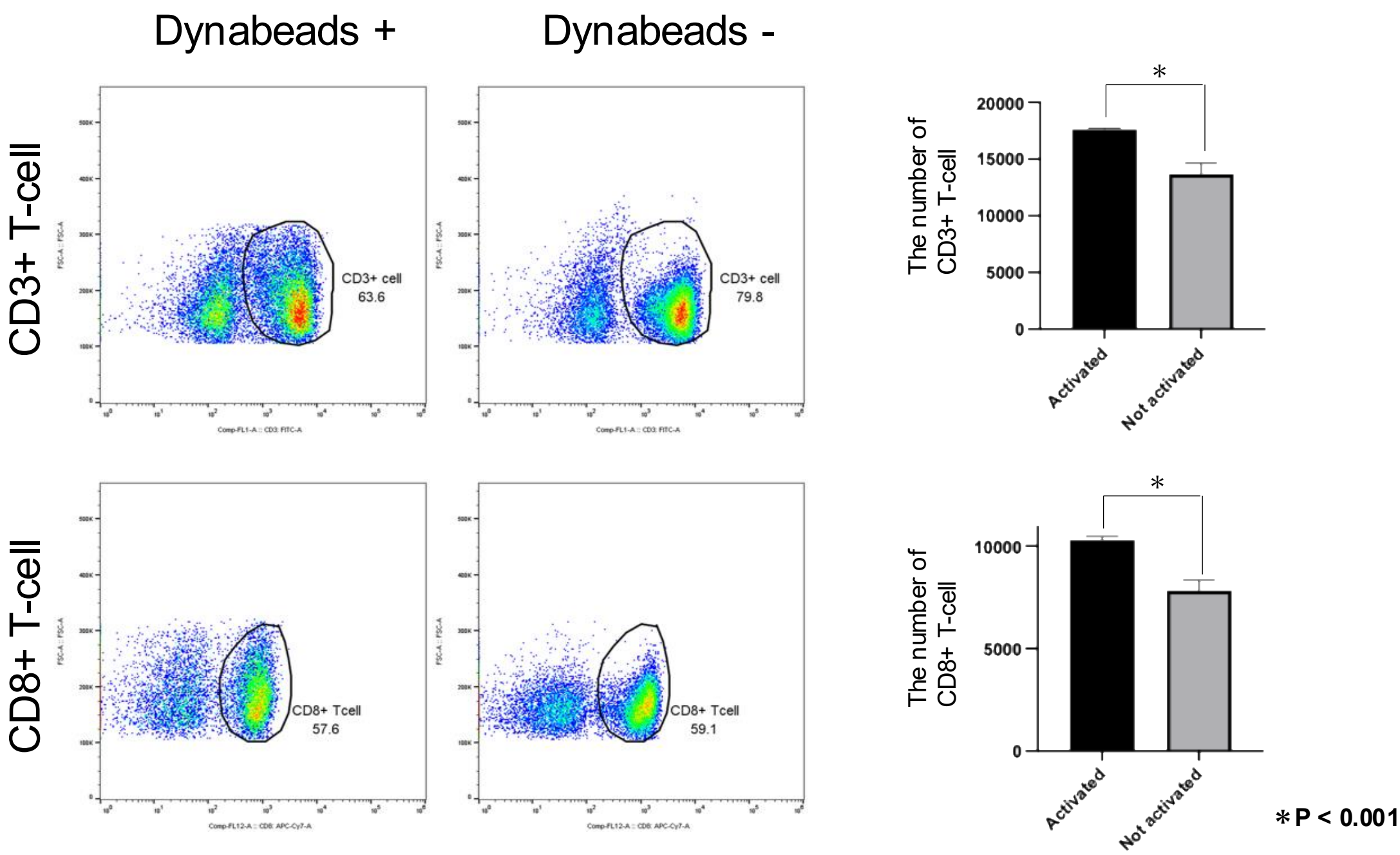

Figure S2

Peripheral blood mononuclear cell-derived T cells were stimulated with anti-CD3/CD28 Dynabeads, and $CD3^+$ and $CD8^+$ T-cell populations were evaluated by flow cytometry before and after stimulation. Anti-CD3/CD28 stimulation increased the absolute numbers of $CD3^+$ and $CD8^+$ T cells, whereas the proportion of $CD8^+$ cells among $CD3^+$ T cells was not markedly altered. These data were used as a quality-control assessment of the activated T-cell population used in the co-culture assay.

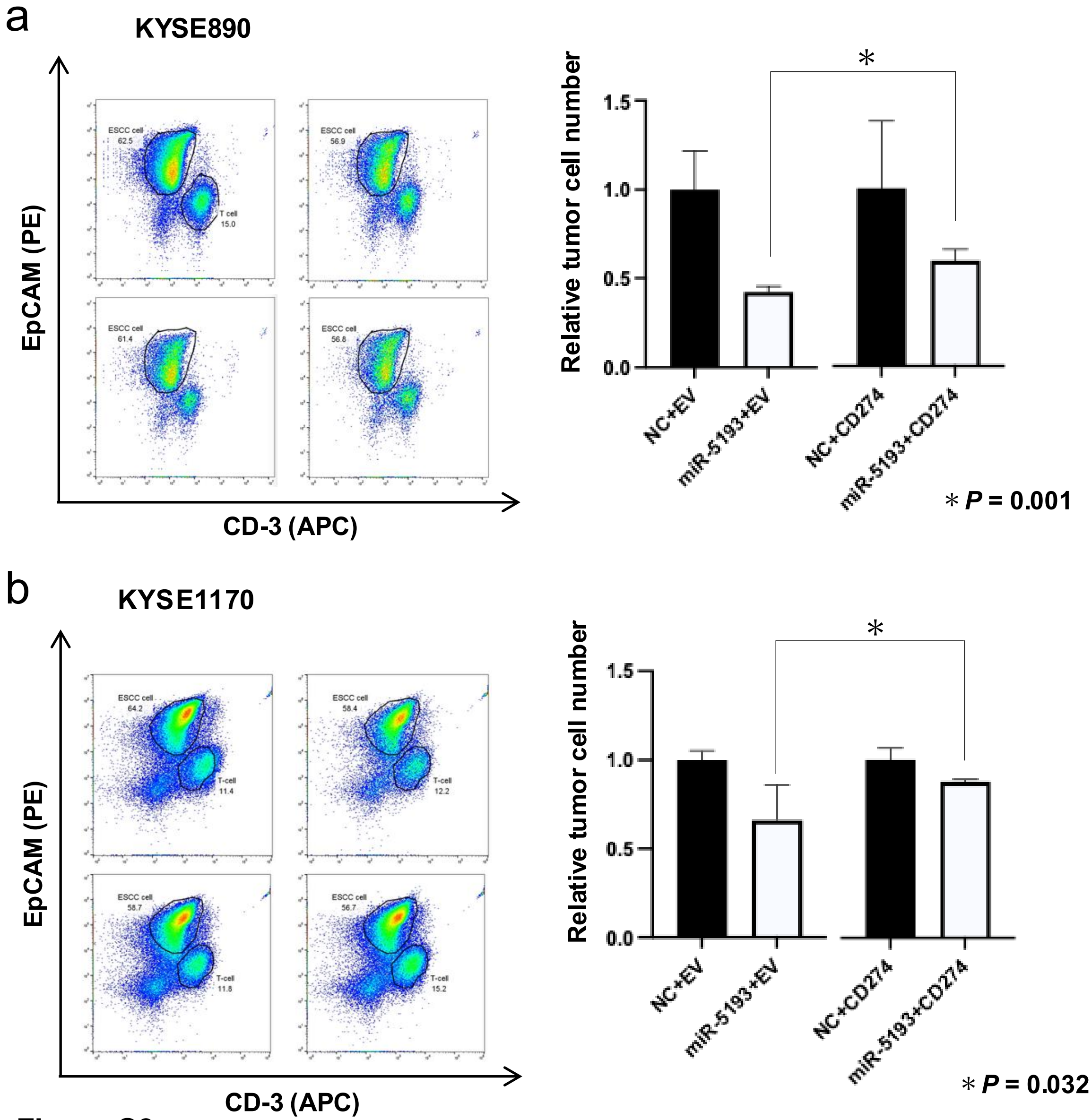

Figure S3

KYSE890 and KYSE1170 cells were transfected with miR-5193 mimic or negative-control mimic, followed by transient transfection with a human CD274/PD-L1 open reading frame expression vector or the corresponding empty vector. The cells were then co-cultured with activated $CD3^+$ T cells. Viable tumor cells were quantified as epithelial cell adhesion molecule-positive, CD3-negative cells by flow cytometry using counting beads. CD274/PD-L1 re-expression partially attenuated the miR-5193 mimic-induced reduction in viable tumor cells, suggesting that miR-5193 enhances T-cell–mediated antitumor activity at least in part through PD-L1 suppression.

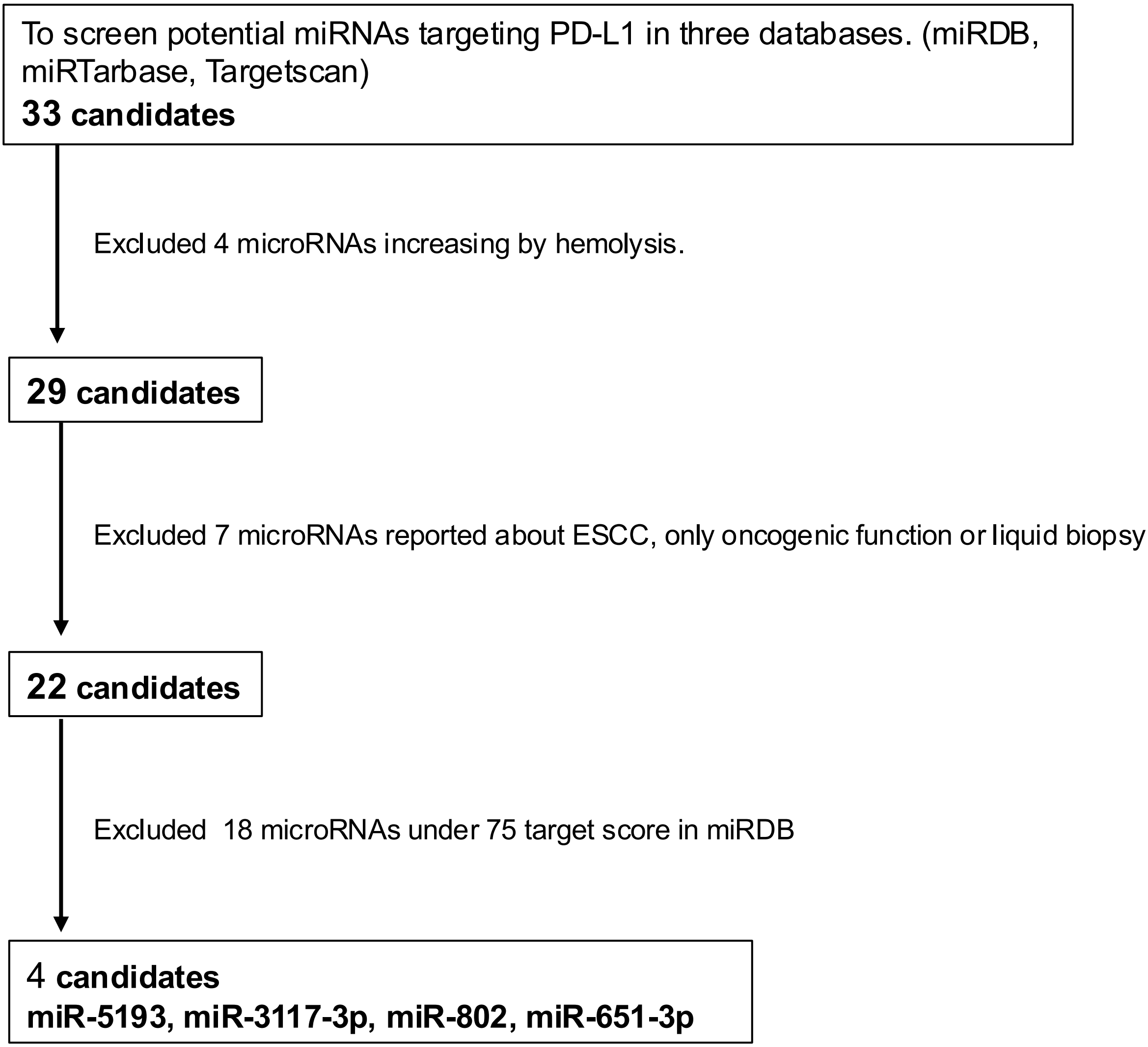

Figure S4

Candidate miRNAs predicted to target PD-L1/CD274 were identified using public miRNA target prediction/validation databases, including miRDB, miRTarBase, and TargetScan. miRNAs supported by multiple databases were prioritized, and candidates previously reported to be hemolysis-related, mainly oncogenic, or already described in upper gastrointestinal cancers were excluded. Four candidate miRNAs, miR-5193, miR-3117-3p, miR-802, and miR-651-3p, were selected for experimental evaluation.
