# Supplementary material for: Low circulating miR-5193 promotes PD-L1-mediated immune evasion and relates to immune checkpoint inhibitor response in esophageal cancer
Source: Mol Ther Nucleic Acids. 2026 Aug 12;37(3):103059. doi: 10.1016/j.omtn.2026.103059 (PMC13542498; doi:10.1016/j.omtn.2026.103059)
Supplement: Supplementary file 2 [file mmc2.pdf]

Molecular Therapy
Nucleic Acids
Original Article

Low circulating miR-5193 promotes PD-L1-mediated immune evasion and relates to immune checkpoint inhibitor response in esophageal cancer

Ryo Ishida,[1,4] Shuhei Komatsu,[1,4] Hajime Kamiya,[1] Takuma Ohashi,[1] Taisuke Imamura,[1] Jun Kiuchi,[1] Keiji Nishibeppu,[1] Yusuke Takashima,[1] Hiroshi Arakawa,[1] Masateru Yamauchi,[1] Satoshi Hamada,[1] Hiroyuki Kanazawa,[1] Hiroki Shimizu,[1] Tomohiro Arita,[1] Toshiyuki Kosuga,[1] Hirotaka Konishi,[1] Hitoshi Fujiwara,[1] Tomoko Iehara,[2] Hitoshi Tsuda,[3] and Atsushi Shiozaki[1]

[1]Division of Digestive Surgery, Department of Surgery, Kyoto Prefectural University of Medicine, 465 Kajii-cho, Kawaramachihirokoji, Kamigyo-ku, Kyoto 602-8566, Japan; [2]Department of Pediatrics, Kyoto Prefectural University of Medicine, 465 Kajii-cho, Kawaramachihirokoji, Kamigyo-ku, Kyoto 602-8566, Japan; [3]Department of Basic Pathology, National Defense Medical College, Tokorozawa, Saitama 359-8513, Japan

Low levels of circulating tumor-suppressor microRNAs are associated with cancer progression and poor prognosis. However, their role in immune evasion and response to immune checkpoint inhibitors (ICIs) remains unclear. We aimed to identify a PD-L1-targeting tumor-suppressor microRNA downregulated in esophageal squamous cell carcinoma (ESCC). Four tumor-suppressor microRNAs (miR-5193, miR-3117-3p, miR-802, and miR-651-3p) predicted to target programmed cell death ligand 1 (PD-L1) were selected. Plasma levels of miR-5193 were significantly lower in ESCC patients than in healthy volunteers. In 122 consecutive ESCC patients, low plasma miR-5193 was associated with advanced tumor depth and pathological stage and was an independent prognostic factor. In ESCC cells, miR-5193 suppressed PD-L1 expression. In a co-culture model of tumor cells and T cells, miR-5193 overexpression enhanced T cell antitumor activity by suppressing PD-L1. Among 56 ESCC patients treated with or without ICIs for recurrence, miR-5193 levels showed a trend toward an inverse association with PD-L1 tumor proportion score. In the low miR-5193 group, disease control rate was higher and ICI-treated patients showed a trend toward longer progression-free survival. Low levels of miR-5193 contribute to ESCC progression and poor outcomes and may serve as a biomarker reflecting tumor immune status and ICI-related outcomes.

INTRODUCTION

Esophageal cancer imposes a substantial global mortality burden and ranks among the leading causes of cancer-related death.[1] Two histologic subtypes are recognized: adenocarcinoma and squamous cell carcinoma, with their distribution varying by region.[2] Adenocarcinoma is the most common histology in Western populations, whereas esophageal squamous cell carcinoma (ESCC) predominates in Asia,[1,3] with five-year survival remaining poor at approximately 20% despite advances in multidisciplinary therapy.[3] In ESCC treatment, immune checkpoint inhibitors (ICIs) targeting the programmed cell death protein 1 (PD-1)-programmed cell death protein 1 ligand (PD-L1) axis have drastically improved outcomes for a subset of patients. However, its clinical response remains limited in patients, and immune-related adverse events occur in over 40% of cases, which limits its broad clinical benefit. These challenges highlight the need to identify biomarkers that can enhance antitumor immunity in ESCC patients.[4,5]

MicroRNAs (miRNAs) are small non-coding RNAs that post-transcriptionally regulate gene expression, in part by binding to the 3′ untranslated regions (3′ UTRs) of target mRNAs. Circulating miRNAs are remarkably stable in blood and enable minimally invasive assessment of tumor biology and host-tumor interactions.[6,7] Multiple miRNAs have been implicated in the regulation of immune checkpoints, including PD-L1, across diverse malignancies.[8–10] However, it remains unclear whether circulating tumor-suppressive miRNAs participate in immune evasion in ESCC, or whether their blood levels can inform ICI-related biology.

Recent studies suggest that neighboring normal cells release tumor-suppressor miRNAs in extracellular vesicles to help maintain tissue homeostasis, while cancer cells may uptake these miRNAs to compensate for intrinsic loss.[11] We therefore hypothesized that as the disease progresses, the supply of these miRNAs declines, circulating levels decrease, and malignant traits intensify. In our previous cancer studies, we reported depletion of tumor-suppressor miRNAs in patient plasma, with low levels of miR-107, miR-655,

Received 17 February 2026; accepted 10 August 2026;
https://doi.org/10.1016/j.omtn.2026.103059.

[4]These authors contributed equally

Correspondence: Shuhei Komatsu MD, PhD, Division of Digestive Surgery, Department of Surgery, Kyoto Prefectural University of Medicine, 465 Kajii-cho, Kawaramachihirokoji, Kamigyo-ku, Kyoto 602-8566, Japan.
E-mail: skomatsu@koto.kpu-m.ac.jp

© 2026 The Author(s). Published by Elsevier Inc. on behalf of The American Society of Gene and Cell Therapy.
This is an open access article under the CC BY license (http://creativecommons.org/licenses/by/4.0/).

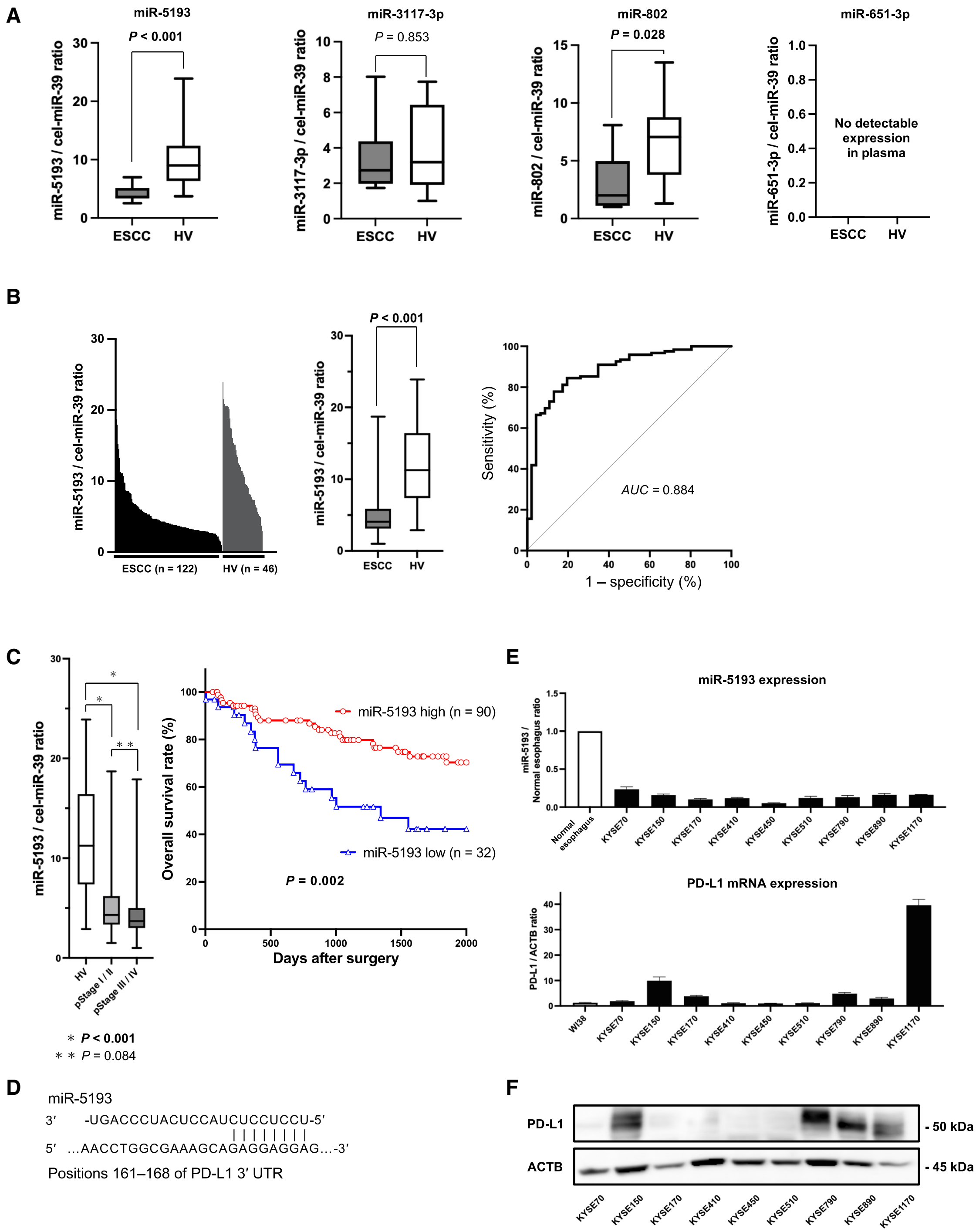

Figure 1. Plasma miR-5193 is downregulated in esophageal squamous cell carcinoma, associates with stage and survival, and targets programmed cell death ligand 1

(A) Pilot screen of candidate microRNAs (miRNAs). Plasma levels of four candidate miRNAs (miR-5193, miR-3117-3p, miR-802, and miR-651-3p) were quantified by quantitative reverse transcription polymerase chain reaction and normalized to cel-miR-39. miR-5193 was significantly lower in esophageal squamous cell carcinoma (ESCC) than in healthy volunteers (HVs), whereas miR-3117-3p showed no difference, and miR-802 showed a modest decrease. miR-651-3p was undetectable in plasma. (B) In the large cohort, waterfall plots and boxplots confirmed lower plasma miR-5193 in ESCC ($n = 122$) compared to HVs ($n = 46$; $p < 0.001$). Receiver operating characteristic analysis indicates robust diagnostic performance (area under the curve = 0.884). (C) Plasma miR-5193 levels decreased with advancing pathological

(legend continued on next page)

and miR-1254 associated with poor prognosis and markers of tumor aggressiveness.[12–14] In this study, we evaluated the clinical and mechanistic relevance of miR-5193 in ESCC to determine its potential as a biomarker and a candidate for nucleic acid immunotherapy.

Through a systematic review of the NCBI and miRNA databases, we focused on tumor-suppressor miR-5193, which targets PD-L1 and is present at low levels in the plasma of ESCC patients. Briefly, we demonstrated that low plasma levels of miR-5193 were significantly associated with advanced pathological stage and served as an independent prognostic factor, reflecting the clinical efficacy of ICIs. Overexpression of miR-5193 in ESCC cells suppressed PD-L1 and enhanced T cell antitumor activity. In gastric cancer, we previously showed that miR-5193 suppresses PD-L1 and increases T cell activity, suggesting a link between circulating RNA networks and tumor immune escape. These findings prompted us to investigate the utility of miR-5193 as both a biomarker and a nucleic acid-based immunotherapy agent for ESCC patients.

RESULTS

Plasma levels of miR-5193 are reduced in patients with ESCC

To identify circulating tumor-suppressive microRNAs, we initially evaluated candidate miRNAs (miR-5193, miR-3117-3p, miR-802, and miR-651-3p) in plasma samples from ESCC patients and healthy volunteers (HVs). miR-651-3p was undetectable in plasma and excluded from further analysis. Quantitative reverse-transcription PCR (RT-qPCR) revealed that plasma miR-5193 levels were significantly lower in ESCC patients compared with HVs ($p < 0.001$), whereas miR-3117-3p showed no significant difference and miR-802 was only slightly decreased ($p = 0.028$) (Figure 1A). Among these candidates, miR-5193 exhibited the most pronounced difference between ESCC and HVs, and therefore became the focus of subsequent analyses. In the expanded cohort (ESCC, $n = 122$; HVs, $n = 46$), plasma miR-5193 remained significantly reduced in ESCC patients ($p < 0.001$) (Figure 1B). Receiver operating characteristic analysis demonstrated that circulating miR-5193 discriminated ESCC patients from HVs with high accuracy (area under the curve = 0.884) (Figure 1B).

Clinical significance of circulating miR-5193 in ESCC

We assessed the relationship between plasma miR-5193 levels and clinicopathological factors in 122 ESCC patients (Table 1). Low plasma miR-5193 expression was significantly associated with advanced tumor depth ($p = 0.009$) and pathological stage ($p = 0.021$). Plasma miR-5193 levels were markedly lower in patients with pathological stage I/II and stage III/IV disease compared with HVs ($p < 0.001$). Moreover, patients with stage III/IV disease tended to have even lower levels than those with stage I/II disease ($p = 0.084$) (Figure 1C). Kaplan-Meier survival analysis showed that patients with low miR-5193 expression ($n = 32$) had significantly poorer overall survival than those with high expression ($n = 90$; $p = 0.002$) (Figure 1C). In multivariable Cox proportional hazards models, low miR-5193 expression remained an independent prognostic factor for overall survival (hazard ratio = 2.05, 95% confidence interval [CI] = 1.1–4.0, $p = 0.033$), together with tumor depth and nodal status (Table 2).

miR-5193 expression is downregulated in ESCC cell lines and predicted to target PD-L1

Bioinformatic analysis identified a putative-binding site for miR-5193 in the 3′ UTR of PD-L1 mRNA (Figure 1D). RT-qPCR revealed markedly reduced miR-5193 expression in all ESCC cell lines compared with normal esophageal tissue (Figure 1E). PD-L1 mRNA expression varied among cell lines, with KYSE1170 exhibiting the highest levels. These findings indicate that ESCC cell lines generally exhibit reduced miR-5193 expression, whereas basal PD-L1 expression is heterogeneous and likely regulated by multiple mechanisms. Consistent with these findings, western blotting confirmed heterogeneous PD-L1 protein expression across ESCC cell lines (Figure 1F). Based on these results, KYSE890 and KYSE1170 cells were selected for subsequent functional assays. To further determine whether miR-5193 regulates PD-L1 through its 3′ UTR, we performed luciferase reporter assays using a pmirGLO vector containing the PD-L1 3′ UTR. In both KYSE890 and KYSE1170 cells, miR-5193 mimic significantly reduced the luciferase activity of the PD-L1 3′ UTR reporter, whereas no significant change was observed with the empty pmirGLO vector (Figure S1). These findings support that miR-5193 regulates PD-L1 through its 3′ UTR.

miR-5193 suppresses PD-L1 expression in ESCC cells

We next examined the effects of miR-5193 on PD-L1 expression *in vitro*. Western blotting showed that PD-L1 protein levels were suppressed by miR-5193 overexpression in KYSE890 and KYSE1170 cells, even after interferon gamma (IFN-γ) stimulation (Figure 2A). Flow cytometry further confirmed a significant reduction in surface PD-L1 in miR-5193-transfected cells compared with negative controls. In KYSE890 cells, the mean fluorescence intensity of PD-L1 decreased significantly under both basal ($p < 0.001$) and IFN-γ-stimulated conditions ($p < 0.001$). Similarly, in KYSE1170 cells, miR-5193 overexpression markedly reduced PD-L1 expression in both basal ($p < 0.001$) and IFN-γ-stimulated conditions ($p < 0.001$) (Figure 2B). These results indicate that miR-5193 strongly suppresses PD-L1 expression on the cell surface, even under cytokine-induced conditions.

stage. Low plasma miR-5193 was associated with poorer overall survival (log rank $p = 0.002$). (D) Programmed cell death ligand 1 (PD-L1) as a direct target of miR-5193. Predicted base-pairing between miR-5193 and the PD-L1 3′ untranslated region (positions 161–168) indicates PD-L1 as a direct target of miR-5193. (E) miR-5193 and PD-L1 mRNA in ESCC cell lines. Compared with normal esophageal epithelium, ESCC cell lines exhibit markedly reduced miR-5193 and variable PD-L1 mRNA expression, with a subset showing high PD-L1 expression. (F) PD-L1 protein across ESCC cell lines. Immunoblotting for PD-L1, using β-actin (ACTB) as a loading control, reflects the mRNA expression pattern.

Table 1. Association between plasma miR-5193 levels and clinicopathological features

Variables		n	Plasma miR-5193 concentration				Univariate[a]
			High (n = 90)		Low (n = 32)		p value
Gender	male	98	73	(81.1%)	25	(78.1%)	0.796
	female	24	17	(18.9%)	7	(21.9%)	
Age	≥65	83	61	(67.8%)	22	(68.8%)	1.000
	<65	39	29	(32.2%)	10	(31.3%)	
Ly[b]	absent	77	59	(65.6%)	18	(56.3%)	0.397
	present	45	31	(34.4%)	14	(43.7%)	
V[c]	absent	65	52	(57.8%)	13	(40.6%)	0.097
	present	57	38	(42.2%)	19	(59.4%)	
T-stage	T1	43	38	(42.2%)	5	(15.6%)	**0.009**
	T2,3,4	79	52	(57.8%)	27	(84.4%)	
N-stage	N0	54	44	(48.9%)	10	(31.3%)	0.099
	N1,2,3	68	46	(51.1%)	22	(68.8%)	
pStage	I	30	27	(30.0%)	3	(9.4%)	**0.021**
	II	43	31	(34.4%)	12	(37.5%)	
	III	45	31	(34.4%)	14	(43.8%)	
	IV	4	1	(1.1%)	3	(9.4%)	

NOTE: significant values are in bold.
[a]p values are from the chi-squared test or Fisher's exact test.
[b]Lymphovascular invasion.
[c]Venous invasion.

miR-5193 enhances T cell-mediated antitumor activity

We then examined whether the suppression of PD-L1 by miR-5193 translates into enhanced immune recognition. Before performing co-culture assays, we confirmed that anti-CD3/CD28 Dynabeads stimulation increased the absolute numbers of $CD3^+$ and $CD8^+$ T cells, while the proportion of $CD8^+$ cells was not markedly altered (Figure S2). Co-culture assays with activated $CD3^+$ T cells revealed that miR-5193 overexpression significantly increased T-cell-mediated cytotoxicity against ESCC cells. Flow cytometry showed a lower proportion of viable $EpCAM^+$ tumor cells in the miR-5193 mimic group compared with the negative control mimic (Figure 2C). In KYSE890 cells, tumor proliferation was significantly reduced in the miR-5193 group when co-cultured with T cells (p = 0.012), with a similar effect observed in KYSE1170 cells (p = 0.047). These results indicate that enforced expression of miR-5193 sensitizes ESCC cells to immune-mediated killing. Additionally, impedance-based real-time analysis confirmed that miR-5193 enhanced T-cell-induced cytotoxicity, as indicated by a decreased normalized cell index relative to controls (Figure 2D). To further examine whether this effect was mediated through PD-L1 suppression, we performed a PD-L1 rescue experiment using a CD274/PD-L1 open reading frame expression vector. CD274/PD-L1 re-expression partially attenuated the miR-5193 mimic-induced reduction in viable tumor cells in both KYSE890 and KYSE1170 cells (Figure S3). These results suggest that miR-5193 enhances T-cell-mediated antitumor activity, at least in part, through PD-L1 suppression.

Clinical relevance of circulating miR-5193 in ESCC patients treated with ICIs

We further investigated the relationship between circulating miR-5193 and ICI efficacy. Among 29 patients treated with ICIs, such as nivolumab or pembrolizumab, plasma miR-5193 levels tended to show a trend toward an inverse association with PD-L1 tumor proportion score (TPS) ($R = -0.227$, p = 0.092) (Figures 3A and 3B). Regarding clinical efficacy after ≥6 weeks of treatment, although ICI efficacy was higher in patients with low miR-5193, the objective response rate (ORR), including partial and complete responses, did not differ between groups (p = 0.201). However, the disease control rate (DCR), including stable disease, partial responses, and complete responses, was significantly higher in patients with low miR-5193 levels than in those with high levels (p = 0.032) (Figure 3C).

DISCUSSION

In this study, low plasma levels of miR-5193 were associated with ESCC progression and poor outcomes. These findings suggest that miR-5193 may represent a promising biomarker candidate and a potential target for future nucleic acid-based immunotherapy targeting PD-L1, building on previous observations in gastric cancer.[15] Specifically, we identified the plasma miR-5193, which was significantly lower in the plasma of ESCC patients compared to HVs. Low levels of plasma miR-5193 were significantly associated with advanced disease stage and poor prognosis. Consistent with prior findings in gastric cancer, we confirmed that overexpression of miR-5193 suppressed PD-L1 expression and enhanced T-cell-mediated

Table 2. Univariate and multivariate analyses of ESCC patient survival using the Cox proportional hazards model

Variables		n	Univariate[a]	Multivariate analysis[b]		
			p value	HR[c]	95% CI[d]	*p* value
Gender	Male vs. female	98 vs. 24	0.415	–	–	–
Age	≥65 vs. <65	83 vs. 39	0.616			
T-stage	T2,3,4 vs. T1	79 vs. 43	**<0.001**	2.97	1.1–7.9	**0.029**
N-stage	N1,2,3 vs. N0	68 vs. 54	**<0.001**	4.07	1.6–10.1	**0.002**
Plasma miR-5193 expression	Low vs. high	32 vs. 90	**<0.001**	2.05	1.1–4.0	**0.033**

NOTE: significant values are in bold.
[a]Univariate survival analysis was performed using the Kaplan-Meier method; significance was determined by log rank test.
[b]Multivariate survival analysis was performed using the Cox proportional hazards model.
[c]HR, hazard ratio.
[d]CI, confidence interval.

antitumor activity in a co-culture model of ESCC cells and T cells *in vitro*. Clinically, our findings show that plasma miR-5193 levels were associated with ICI outcomes in ESCC, with greater disease control during ICI therapy in the miR-5193-low group. Plasma miR-5193 also showed an inverse relationship with tumor PD-L1 expression, supporting its potential as a biomarker that reflects prognosis and PD-L1-related tumor immune status. These clinical and functional findings support a model in which reduced circulating miR-5193 is associated with PD-L1-related immune evasion in ESCC, while restoration of miR-5193 suppresses tumor-cell PD-L1 and enhances T-cell-mediated antitumor activity.

miRNAs are known to originate not only from cell lysis but also from active secretion, serving as mediators of intercellular communication. Our findings suggest that low levels of the tumor-suppressive miR-5193 contribute to immune evasion mechanisms, leading to PD-L1 overexpression. However, in the context of low blood levels of miR-5193 and increased PD-L1, the miR-5193–PD-L1 axis could represent an Achilles' heel and a promising target for checkpoint blockade therapies. Conversely, high plasma levels of miR-5193 may reduce PD-L1 expression and support T cell function. This therapeutic paradox aligns with prior studies showing that PD-L1 expression on tumor and host immune cells can be required for effective blockade and that experimentally increasing PD-L1 can sensitize tumors to anti-PD-L1 therapy.[16,17] Although recent studies have identified multiple factors that regulate tumor PD-L1—including IFN-γ, JAK/STAT, and IRF1 signaling,[18–21] post-translational stabilization via CMTM6,[22] and epigenetic control[23,24]—circulating tumor-suppressive miRNAs, such as miR-5193, may also play a regulatory role in the cancer immune system.

While previous studies have demonstrated that several miRNAs can regulate PD-L1 expression in tumor cells, their clinical relevance to ICI therapy remains unclear. In this study, we investigated whether plasma miR-5193 levels were associated not only with tumor PD-L1 expression but also with the clinical efficacy of ICI in both short- and long-term outcomes in ESCC patients with recurrences (Figure 3). Specifically, 56 patients with recurrent ESCC were included in this analysis. Among them, 29 received ICIs, including 24 who received ICIs for reccurent disease. Plasma miR-5193 levels showed a non-significant inverse correlation with tumor TPS, a clinical PD-L1 expression scoring system, suggesting that circulating miR-5193 may reflect tumor immune status. Regarding treatment response at ≥6 weeks after ICI initiation, the DCR was significantly higher in the low-miR-5193 group than in the high-miR-5193 group. In long-term outcomes, patients with low plasma miR-5193 who received ICIs had better progression-free survival (PFS) than those who did not. In the high-blood miR-5193 group, PFS was favorable and similar regardless of ICI treatment (Figure 3D). These findings suggest that plasma miR-5193 levels are associated with ICI-related outcomes and prognosis in ESCC patients with recurrence. However, these observations should be interpreted cautiously, as the analysis of ICI-treated patients was exploratory and involved a limited cohort size. Thus, patients with low miR-5193, indicating high PD-L1, may derive differential clinical benefit from ICIs, whereas those with high miR-5193, indicating low PD-L1, may require alternative strategies. This approach could form the basis of a miRNA biomarker-guided treatment strategy in ESCC.

The most striking finding in this study is that our results suggest potential treatment strategies tailored to miR-5193 status. Developing delivery systems for tumor-suppressor miRNAs and restoring blood miR-5193 in the tumor microenvironment through nucleic acid therapy might reduce PD-L1 expression and enhance T cell activity. In *in vivo* models, we previously demonstrated that restoring plasma levels of tumor-suppressive miRNAs, such as miR-107 and miR-655, through subcutaneous injection to levels observed in healthy individuals suppressed tumor progression without significant adverse events.[12,13] More recently, we showed in gastric cancer that restoring miR-5193 suppressed PD-L1 expression and enhanced antitumor activity.[15] These findings support the potential utility of miR-5193 as a biomarker candidate and a therapeutic target.

Our findings demonstrate that plasma miR-5193 is reduced in ESCC patients and is associated with prognosis and PD-L1-related tumor immune status. In our previous *in vivo* study of miR-5193 in gastric

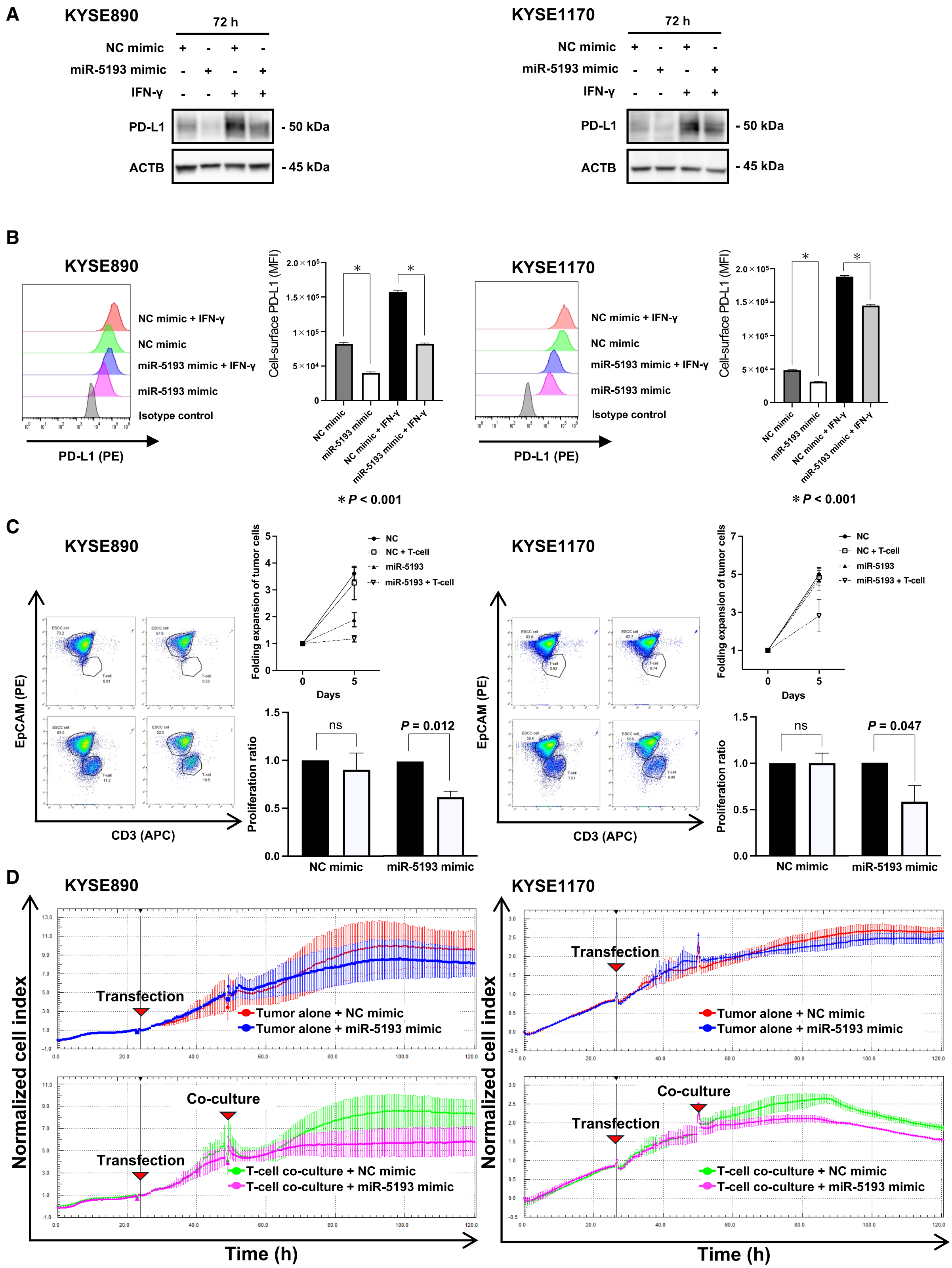

(legend on next page)

cancer, subcutaneous administration enhanced T cell tumor-killing activity and trafficking in mice. Therefore, in the present study, we focused on the clinical relevance of plasma miR-5193 in ESCC, particularly in relation to PD-L1 expression and ICI-related outcomes in a real-world clinical setting. Nevertheless, several limitations should be acknowledged. Because the ICI-treated cohort was limited, the association between plasma miR-5193 and ICI-related outcomes should be interpreted as exploratory and requires validation in larger prospective cohorts. In addition, this study lacked *in vivo* validation in ESCC models. Circulating miR-5193 may also originate from multiple cellular sources, including non-tumor cells and extracellular vesicle-mediated secretion, and may not exclusively reflect tumor-derived miRNA. Furthermore, although $CD3^+$ and $CD8^+$ T cell populations were assessed as quality-control measures, detailed phenotyping of $CD4^+$ T cell subsets and $FoxP3^+CD25^+$ regulatory T cells was not performed. Further studies are needed to clarify the cellular origin, secretion mechanisms, physiological effects, and therapeutic potential of miR-5193 in ESCC.

MATERIALS AND METHODS

Ethics approval and consent to participate

All experimental methods were carried out in accordance with relevant guidelines and regulations, such as the Declaration of Helsinki. Written informed consent was obtained from all patients to use their tissue specimens and blood samples. This study was approved by the institutional review boards of Kyoto Prefectural University of Medicine (ERB-C-67).

Patient samples

Peripheral blood was collected from ESCC patients before treatment and from HVs with no history of malignant disease. All patients included in this cohort had histologically confirmed esophageal squamous cell carcinoma, and no patients with esophageal adenocarcinoma were included. Samples were processed immediately after collection. Plasma was separated using sequential centrifugation (1,500 rpm for 30 min, 3,000 rpm for 5 min, and 4,500 rpm for 5 min) to minimize contamination by cellular nucleic acids. The plasma supernatant was stored at -80°C until RNA extraction. Clinicopathological data were recorded according to the Union for International Cancer Control TNM classification.

Systematic selection of candidate miRNAs

We conducted a systematic search of NCBI PubMed and miRNA databases to identify microRNAs predicted to bind the 3′ UTR of PD-L1. Target prediction algorithms (miRDB, TargetScan, and miRTarBase) were used. Candidates previously reported as oncogenic, as well as those upregulated by hemolysis or already described in upper gastrointestinal cancers, were excluded (Figure S4). Four candidates were ultimately selected: miR-5193, miR-3117-3p, miR-802, and miR-651-3p. Among these, miR-5193 was prioritized based on high prediction scores and because it showed the most pronounced reduction in plasma samples from ESCC patients compared with HVs.

RNA extraction and quantification of plasma miRNAs

Plasma samples (400 μL) were processed for RNA isolation using the miRVana PARIS Kit (Ambion, Austin, TX, USA) according to the manufacturer's instructions, and RNA was eluted in 100 μL of preheated buffer. A synthetic spike-in control (cel-miR-39; QIAGEN, Hilden, Germany) was added to each sample prior to extraction for technical normalization. RT-qPCR was performed using TaqMan MicroRNA Assays (Applied Biosystems, Foster City, CA, USA) on a Step OnePlus Real-Time PCR system. The assays used were hsa-miR-5193 (assay ID: 476857), hsa-miR-651-3p (assay ID: 466254_mat), hsa-miR-802 (assay ID: MC11813), and hsa-miR-3117-3p (assay ID: 241504_mat). Relative expression levels were calculated using the $2^{-\Delta\Delta Ct}$ method, with cel-miR-39 as the reference for plasma samples and U6 snRNA (RNU6B) for human tissue and cultured cell samples.[13–15,25–29]

Cell culture and transfection

Human ESCC cell lines (KYSE series; Japanese Collection of Research Bioresources, Osaka, Japan) were cultured in RPMI-1640 supplemented with 10% fetal bovine serum at 37°C with 5% CO_2. Cells were authenticated by short tandem repeat profiling and confirmed to be mycoplasma-free. miR-5193 mimic (assay ID: MC23346, Ambion) and negative control mimic were transfected at a final concentration of 12 nM using Lipofectamine RNAiMAX (Invitrogen, Carlsbad, CA, USA).

Luciferase reporter assay

The predicted miR-5193 binding sequence in the PD-L1/CD274 3′ UTR was identified using miRDB, TargetScan, and miRTarBase. The PD-L1 3′UTR fragment was cloned downstream of the firefly luciferase gene in the pmirGLO Dual-Luciferase miRNA Target Expression Vector (Promega, Madison, WI, USA) and verified by sequencing. KYSE890 and KYSE1170 cells were co-transfected with the empty pmirGLO vector or pmirGLO-PD-L1 3′ UTR

Figure 2. miR-5193 reduces PD-L1 in ESCC cells and enhances T cell antitumor activity

(A) PD-L1 protein levels after miR-5193 overexpression ± interferon gamma (IFN-γ). KYSE890 and KYSE1170 cells were transfected with a miR-5193 mimic or negative-control (NC) mimic for 72 h and stimulated with IFN-γ during the final 48 h, as indicated. Immunoblotting shows that miR-5193 reduces PD-L1 at baseline and after IFN-γ stimulation. (B) Representative histograms and mean fluorescence intensity (MFI) quantification demonstrate decreased PD-L1 on the cell surface after miR-5193 overexpression, both at baseline and under IFN-γ stimulation, in KYSE890 and KYSE1170 cells ($p < 0.001$). (C) T cell co-culture assay. ESCC cells were co-cultured with activated human T cells. Flow cytometry (CD3-allophycocyanin [APC] vs. epithelial cell adhesion molecule [EpCAM]-phycoerythrin [PE]) demonstrates a reduction in the fraction of $EpCAM^+$ tumor cells over time when tumor cells overexpress miR-5193 (day 1 → day 5), with decreased tumor-cell proliferation ratios (KYSE890: $p = 0.012$; KYSE1170: $p = 0.047$; ns, not significant). (D) Impedance-based tumor-cell killing assay. Real-time cell analysis shows that miR-5193 overexpression modestly restricts tumor growth in monoculture and, in T cell co-culture, accelerates the decline of the normalized cell index, indicating enhanced T-cell-mediated cytotoxicity.

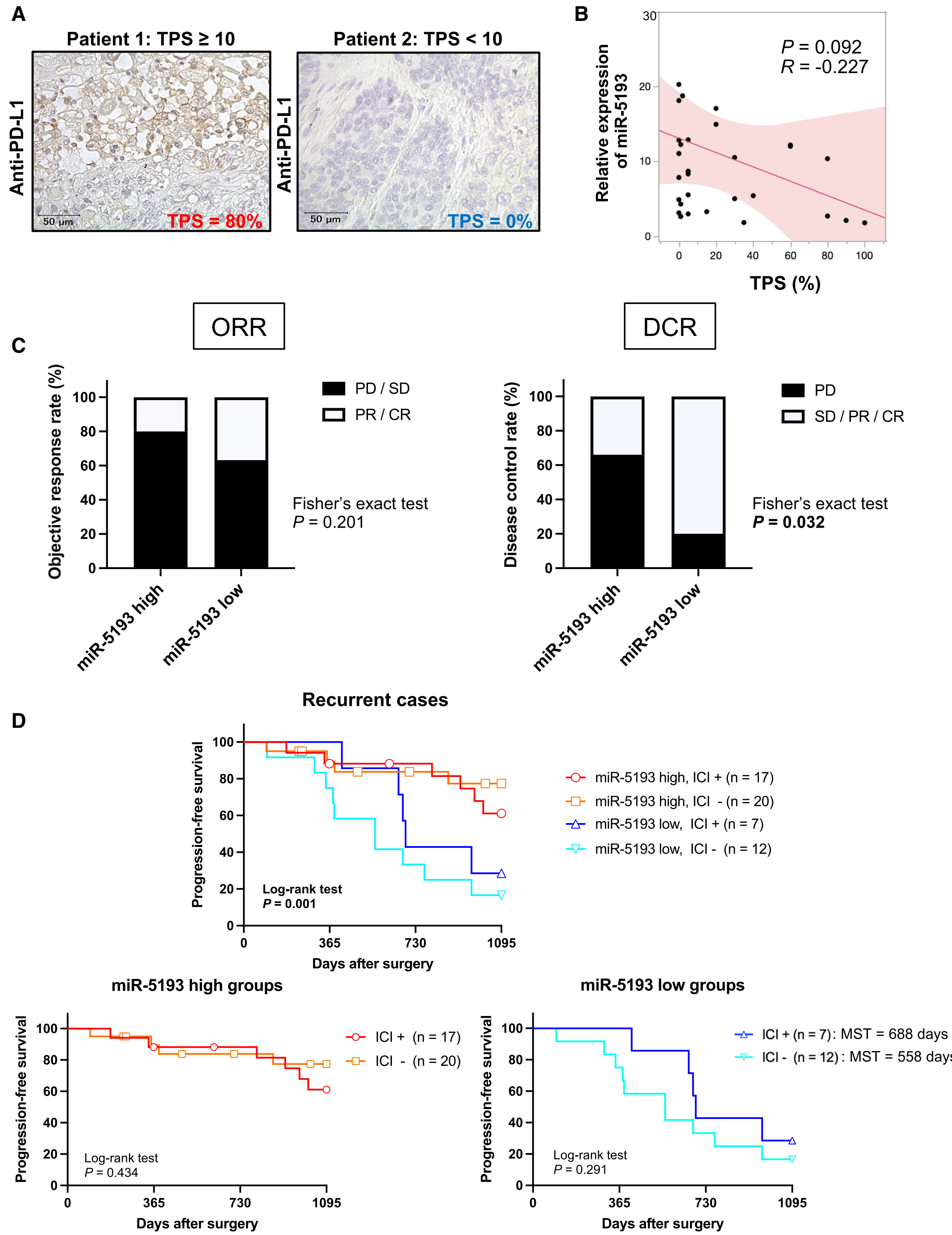

Figure 3. Circulating miR-5193 inversely correlates with tumor PD-L1 and associates with ICI outcomes in ESCC

(A) PD-L1 protein expression was evaluated using an anti-PD-L1 antibody and scored by the tumor proportion score (TPS). Patient 1 shows strong membranous PD-L1 staining in tumor cells with a TPS of 80% (high expression, TPS ≥10), whereas patient 2 shows no detectable PD-L1 staining with a TPS of 0% (low expression, TPS <10). (B) Relationship between plasma miR-5193 (normalized to cel-miR-39) and tumor PD-L1 (TPS) in 29 ESCC patients receiving PD-1 therapy. An inverse trend is observed (Spearman $R = -0.227$, $p = 0.092$); the line indicates the fitted trend with 95% confidence interval shading. (C) Immune checkpoint inhibitor (ICI) efficacy stratified by plasma miR-5193. Patients were divided into miR-5193-high ($n = 9$) and miR-5193-low ($n = 20$) groups. The cutoff value was determined using the minimum p-value method

(legend continued on next page)

construct together with miR-5193 mimic or negative-control mimic using Lipofectamine 3000 with P3000 reagent (Invitrogen, Carlsbad, CA, USA). After 48 h, firefly and Renilla luciferase activities were measured using the Dual-Glo Luciferase Reporter Assay System (Promega). Firefly activity was normalized to Renilla activity, and the relative luciferase activity of the negative-control mimic was set to 1 for each reporter construct.

Western blotting

Anti-PD-L1 rabbit monoclonal antibody (13684; Cell Signaling Technology, Danvers, MA, USA) and anti-β-actin (ACTB) rabbit monoclonal antibody (3700; Cell Signaling Technology) were used. Cells were lysed, and proteins were extracted using M-PER® Mammalian Protein Extraction Reagent (Thermo Fisher Scientific, Waltham, MA, USA).

IFN-γ stimulation and PD-L1 detection

Twenty-four hours after transfection, tumor cells were treated with 10 ng/mL recombinant human IFN-γ (PeproTech, Rocky Hill, NJ, USA) for 48 h to induce PD-L1 expression. Total PD-L1 protein was analyzed by western blotting using anti-PD-L1 antibody (Cell Signaling Technology) and normalized to β-actin. For surface PD-L1 detection, cells were stained with phycoerythrin (PE)-conjugated anti-PD-L1 antibody (BioLegend, San Diego, CA, USA) and analyzed by flow cytometry (BD Accuri C6 Plus) (BD Biosciences, San Jose, CA, USA).

T cell isolation and co-culture assays

Peripheral blood mononuclear cells from HVs were isolated by density-gradient centrifugation using Lymphocyte Separation Medium 1077 (Fujifilm Wako, Osaka, Japan). $CD3^+$ T cells were activated with anti-cluster of differentiation 3 (CD3)/CD28-coated magnetic beads (Dynabeads Human T-Activator, Invitrogen) and cultured in TexMACS medium (Miltenyi Biotec, Bergisch Gladbach, Germany) supplemented with 200 IU/mL interleukin-2 for at least 7 days. For co-culture experiments, tumor cells transfected with miRNA mimics were seeded in 6-well plates and co-cultured with activated T cells at a 1:3 tumor-to-T cell ratio. For PD-L1 rescue experiments, tumor cells were first transfected with miRNA mimics and then, 24 h later, transiently transfected with a human CD274/PD-L1 open reading frame expression vector, pRP[Exp]-EGFP/Puro-EF1A > hCD274, or the corresponding empty control vector, pRP[Exp]-EGFP/Puro-EF1A > ORF_Stuffer (VectorBuilder, Chicago, IL, USA), using Lipofectamine 3000 with P3000 reagent. After 72 h, cells were harvested and stained with anti-epithelial cell adhesion molecule (EpCAM) and anti-CD3 antibodies to distinguish tumor cells from T cells. Viable $EpCAM^+$ tumor cells were quantified by flow cytometry and normalized using counting beads (Thermo Fisher Scientific). This procedure followed previously reported methods, with minor modifications.[15]

Flow cytometry

To assess cell-surface molecules, ESCC cells were stained with PE-conjugated anti-PD-L1 antibody (BioLegend). EpCAM expression was detected using PE-conjugated anti-EpCAM antibody (BioLegend). T cells were identified with allophycocyanin (APC)-conjugated anti-CD3 antibody (BioLegend). Data were acquired on a BD Accuri C6 Plus cytometer and analyzed using FlowJo software (Tree Star, Ashland, OR, USA).

In vitro tumor cell-killing assay

ESCC cells were seeded in 6-well plates at 1×10^5 cells/well and transfected with either miR-5193 mimic or negative control mimic. After 24 h, anti-CD3/CD28 bead-stimulated T cells were added at a 1:3 ratio (tumor:T cells). Following 72 h of co-culture, the mixed cell population was stained with PE-anti-EpCAM and APC-anti-CD3 to distinguish tumor cells from T cells. CountBright Absolute Counting Beads (Thermo Fisher Scientific) were included, and viable tumor cells ($EpCAM^+$, $CD3^-$ cell fraction) were quantified by flow cytometry. Relative tumor proliferation was calculated by normalizing to tumor cells cultured without T cells. The assay was adapted from Kamiya et al.[15] with minor modifications.

Impedance-based killing assay

Real-time cytotoxicity was measured using the xCELLigence RTCA DP system (ACEA Biosciences, San Diego, CA, USA). ESCC cells (5×10^3/well) were seeded on E-plate 16 (ACEA Biosciences) and allowed to adhere for 24 h before transfection with miR-5193 mimic or negative control mimic (12 nM). Twenty-four hours post-transfection, activated T cells (2×10^4/well) were added. Impedance was monitored continuously, and normalized cell index values were calculated using RTCA software version 2.0 (ACEA Biosciences), following the method described by Kamiya et al.[15]

Patients receiving ICIs

Plasma samples from patients with ESCC treated with nivolumab or pembrolizumab at our institution between 2020 and 2024 (n = 51) were retrospectively analyzed. Seven patients were excluded due to insufficient plasma, and 15 patients who discontinued therapy within 60 days were also excluded, leaving 29 patients for analysis. Tumor

to identify the most discriminative threshold for objective response rate (ORR) and disease control rate (DCR). Stacked bars show ORR (complete response [CR] + partial response [PR]) and DCR (CR + PR + stable disease [SD]). ORR did not differ significantly between groups (p = 0.201), whereas DCR was higher in the miR-5193-low group (p = 0.032). (D) Kaplan-Meier curves showing progression-free survival (PFS) among patients with recurrent ESCC stratified by miR-5193 expression and ICI treatment. Recurrent cases were divided into four groups: high-miR-5193 expression with ICI treatment (red line, n = 17), high miR-5193 without ICI treatment (orange line, n = 20), low miR-5193 with ICI treatment (blue line, n = 7), and low miR-5193 without ICI treatment (light blue line, n = 12). The cutoff for miR-5193 expression was the same as in Figure 1C. PFS differed significantly among the four groups (log rank p = 0.001). In the high miR-5193 group, there was no significant difference in PFS between patients with and without ICI treatment (p = 0.434). In the low miR-5193 group, the median survival time (MST) was 688 days in ICI-treated patients (n = 7) and 558 days in untreated patients (n = 12), with a trend toward longer PFS in the ICI-treated group (p = 0.291).

PD-L1 expression was assessed by immunohistochemistry, and the TPS was recorded. Radiologic responses were evaluated at ≥6 weeks after the initiation of therapy, according to RECIST v.1.1. Patients were stratified into high- and low-miR-5193 groups, and DCR was compared between the two groups.

Immunohistochemistry

Tumor samples were fixed in 10% neutral-buffered formalin, embedded in paraffin, and stored at room temperature. Sections were prepared and stained within two weeks. After deparaffinization, antigen retrieval was performed by heating the sections in 10 mmol/L citrate buffer (pH 9.0) at 95°C for 60 min. Endogenous peroxidase activity was quenched with 3% hydrogen peroxide for 20 min. Slides were then incubated with a protein-blocking reagent (Block Ace; Dainippon Sumitomo Pharmaceutical, Osaka, Japan) for 30 min at room temperature, followed by a 60-min exposure to rabbit monoclonal anti-PD-L1 antibody (Cell Signaling Technology; 1:500). After washing with PBS, signals were visualized using the EnVision+ HRP Detection System (Dako, Carpinteria, CA, USA) with diaminobenzidine as the chromogen, and nuclei were counterstained with Mayer's hematoxylin. PD-L1 expression was scored using TPS, with TPS <10 defined as low expression and TPS ≥10 as high expression.

Statistical analysis

Continuous variables were compared using Student's *t* test or the Mann-Whitney U test, as appropriate. Categorical variables were analyzed with the chi-squared test or Fisher's exact test. Survival was estimated by the Kaplan-Meier method and compared using the log rank test. Multivariable analyses were performed with Cox proportional hazards models. Associations between plasma miRNA levels and tumor PD-L1 TPS were assessed using Spearman's rank correlation. All tests were two-sided, and $p < 0.05$ was considered statistically significant. Analyses were performed using JMP Pro 16.

DATA AND CODE AVAILABILITY

The datasets generated and/or analyzed during the current study are not publicly available due to the personal information protection law in Japan but are available after the permission from the institutional review board and the corresponding author on reasonable request.

ACKNOWLEDGMENTS

Not applicable to this study.

AUTHOR CONTRIBUTIONS

R.I. and S.K. designed, and S.K. and A.S. reviewed the research; R.I., H. Kamiya, H.A., Y.T., K.N., M.Y., S.H. and H. Kanazawa performed cell culture and molecular biology experiments; S.K., T.O., T.I., J.K., K.N., H.S., T.A., T.K., H. Konishi, H.F., T.I., H.T., and A.S. provided clinical specimens and performed the clinical data analyses. R.I. and S.K. wrote the paper. All authors read and approved the final manuscript.

DECLARATION OF INTERESTS

The authors declare that they have no competing interests.

DECLARATION OF GENERATIVE AI AND AI-ASSISTED TECHNOLOGIES IN THE WRITING PROCESS

During the preparation of this manuscript, the authors used ChatGPT (OpenAI) to assist with language editing. The authors reviewed and edited the content as needed and take full responsibility for the content of the publication.

SUPPLEMENTAL INFORMATION

Supplemental information can be found online at https://doi.org/10.1016/j.omtn.2026.103059.

REFERENCES

1. Bray, F., Laversanne, M., Sung, H., Ferlay, J., Siegel, R.L., Soerjomataram, I., and Jemal, A. (2024). Global cancer statistics 2022: GLOBOCAN estimates of incidence and mortality worldwide for 36 cancers in 185 countries. CA Cancer J. Clin. *74*, 229–263. https://doi.org/10.3322/caac.21834.
2. Siewert, J.R., and Ott, K. (2007). Are squamous and adenocarcinomas of the esophagus the same disease? Semin. Radiat. Oncol. *17*, 38–44. https://doi.org/10.1016/j.semradonc.2006.09.007.
3. Lagergren, J., Smyth, E., Cunningham, D., and Lagergren, P. (2017). Oesophageal cancer. Lancet *390*, 2383–2396. https://doi.org/10.1016/S0140-6736(17)31462-9.
4. Kato, K., Cho, B.C., Takahashi, M., Okada, M., Lin, C.Y., Chin, K., Kadowaki, S., Ahn, M.J., Hamamoto, Y., Doki, Y., et al. (2019). Nivolumab versus chemotherapy in patients with advanced oesophageal squamous cell carcinoma refractory or intolerant to previous chemotherapy (ATTRACTION-3): a multicentre, randomised, open-label, phase 3 trial. Lancet Oncol. *20*, 1506–1517. https://doi.org/10.1016/S1470-2045(19)30626-6.
5. Sun, J.M., Shen, L., Shah, M.A., Enzinger, P., Adenis, A., Doi, T., Kojima, T., Metges, J.P., Li, Z., Kim, S.B., et al. (2021). Pembrolizumab plus chemotherapy versus chemotherapy alone for first-line treatment of advanced oesophageal cancer (KEYNOTE-590): a randomised, placebo-controlled, phase 3 study. Lancet *398*, 759–771. https://doi.org/10.1016/S0140-6736(21)01234-4.
6. Di Leva, G., Garofalo, M., and Croce, C.M. (2014). MicroRNAs in cancer. Annu. Rev. Pathol. *9*, 287–314. https://doi.org/10.1146/annurev-pathol-012513-104715.
7. Arroyo, J.D., Chevillet, J.R., Kroh, E.M., Ruf, I.K., Pritchard, C.C., Gibson, D.F., Mitchell, P.S., Bennett, C.F., Pogosova-Agadjanyan, E.L., Stirewalt, D.L., et al. (2011). Argonaute2 complexes carry a population of circulating microRNAs independent of vesicles in human plasma. Proc. Natl. Acad. Sci. USA *108*, 5003–5008. https://doi.org/10.1073/pnas.1019055108.
8. Ashizawa, M., Okayama, H., Ishigame, T., Thar Min, A.K., Saito, K., Ujiie, D., Murakami, Y., Kikuchi, T., Nakayama, Y., Noda, M., et al. (2019). miRNA-148a-3p Regulates Immunosuppression in DNA Mismatch Repair-Deficient Colorectal Cancer by Targeting PD-L1. Mol. Cancer Res. *17*, 1403–1413. https://doi.org/10.1158/1541-7786.MCR-18-0831.
9. Miliotis, C., and Slack, F.J. (2021). miR-105-5p regulates PD-L1 expression and tumor immunogenicity in gastric cancer. Cancer Lett. *518*, 115–126. https://doi.org/10.1016/j.canlet.2021.05.037.
10. Yadav, R., Khatkar, R., Yap, K.C.H., Kang, C.Y.H., Lyu, J., Singh, R.K., Mandal, S., Mohanta, A., Lam, H.Y., Okina, E., et al. (2024). The miRNA and PD-1/PD-L1 signaling axis: an arsenal of immunotherapeutic targets against lung cancer. Cell Death Discov. *10*, 414. https://doi.org/10.1038/s41420-024-02182-1.
11. Kosaka, N., Iguchi, H., Yoshioka, Y., Hagiwara, K., Takeshita, F., and Ochiya, T. (2012). Competitive interactions of cancer cells and normal cells via secretory microRNAs. J. Biol. Chem. *287*, 1397–1405. https://doi.org/10.1074/jbc.M111.288662.
12. Imamura, T., Komatsu, S., Ichikawa, D., Miyamae, M., Okajima, W., Ohashi, T., Kiuchi, J., Nishibeppu, K., Konishi, H., Shiozaki, A., et al. (2017). Depleted tumor suppressor miR-107 in plasma relates to tumor progression and is a novel therapeutic target in pancreatic cancer. Sci. Rep. *7*, 5708. https://doi.org/10.1038/s41598-017-06137-8.
13. Kiuchi, J., Komatsu, S., Imamura, T., Nishibeppu, K., Shoda, K., Arita, T., Kosuga, T., Konishi, H., Shiozaki, A., Okamoto, K., et al. (2019). Low levels of tumour suppressor miR-655 in plasma contribute to lymphatic progression

and poor outcomes in oesophageal squamous cell carcinoma. Mol. Cancer *18*, 2. https://doi.org/10.1186/s12943-018-0929-3.

14. Takashima, Y., Komatsu, S., Ohashi, T., Kiuchi, J., Nishibeppu, K., Kamiya, H., Arakawa, H., Ishida, R., Shimizu, H., Arita, T., et al. (2023). Plasma miR-1254 as a predictive biomarker of chemosensitivity and a target of nucleic acid therapy in esophageal cancer. Cancer Sci. *114*, 3027–3040. https://doi.org/10.1111/cas.15830.
15. Kamiya, H., Komatsu, S., Takashima, Y., Ishida, R., Arakawa, H., Nishibeppu, K., Kiuchi, J., Imamura, T., Ohashi, T., Shimizu, H., et al. (2024). Low blood level of tumour suppressor miR-5193 as a target of immunotherapy to PD-L1 in gastric cancer. Br. J. Cancer *130*, 671–681. https://doi.org/10.1038/s41416-023-02532-3.
16. Lau, J., Cheung, J., Navarro, A., Lianoglou, S., Haley, B., Totpal, K., Sanders, L., Koeppen, H., Caplazi, P., McBride, J., et al. (2017). Tumour and host cell PD-L1 is required to mediate suppression of anti-tumour immunity in mice. Nat. Commun. *8*, 14572. https://doi.org/10.1038/ncomms14572.
17. Jiao, S., Xia, W., Yamaguchi, H., Wei, Y., Chen, M.K., Hsu, J.M., Hsu, J.L., Yu, W.H., Du, Y., Lee, H.H., et al. (2017). PARP Inhibitor Upregulates PD-L1 Expression and Enhances Cancer-Associated Immunosuppression. Clin. Cancer Res. *23*, 3711–3720. https://doi.org/10.1158/1078-0432.CCR-16-3215.
18. Lin, X., Kang, K., Chen, P., Zeng, Z., Li, G., Xiong, W., Yi, M., and Xiang, B. (2024). Regulatory mechanisms of PD-1/PD-L1 in cancers. Mol. Cancer *23*, 108. https://doi.org/10.1186/s12943-024-02023-w.
19. Zaretsky, J.M., Garcia-Diaz, A., Shin, D.S., Escuin-Ordinas, H., Hugo, W., Hu-Lieskovan, S., Torrejon, D.Y., Abril-Rodriguez, G., Sandoval, S., Barthly, L., et al. (2016). Mutations Associated with Acquired Resistance to PD-1 Blockade in Melanoma. N. Engl. J. Med. *375*, 819–829. https://doi.org/10.1056/NEJMoa1604958.
20. Ebine, K., Kumar, K., Pham, T.N., Shields, M.A., Collier, K.A., Shang, M., DeCant, B.T., Urrutia, R., Hwang, R.F., Grimaldo, S., et al. (2018). Interplay between interferon regulatory factor 1 and BRD4 in the regulation of PD-L1 in pancreatic stellate cells. Sci. Rep. *8*, 13225. https://doi.org/10.1038/s41598-018-31658-1.
21. Garcia-Diaz, A., Shin, D.S., Moreno, B.H., Saco, J., Escuin-Ordinas, H., Rodriguez, G.A., Zaretsky, J.M., Sun, L., Hugo, W., Wang, X., et al. (2017). Interferon Receptor Signaling Pathways Regulating PD-L1 and PD-L2 Expression. Cell Rep. *19*, 1189–1201.
22. Burr, M.L., Sparbier, C.E., Chan, Y.C., Williamson, J.C., Woods, K., Beavis, P.A., Lam, E.Y.N., Henderson, M.A., Bell, C.C., Stolzenburg, S., et al. (2017). CMTM6 maintains the expression of PD-L1 and regulates anti-tumour immunity. Nature *549*, 101–105. https://doi.org/10.1038/nature23643.
23. Wu, Y., Zhou, L., Zou, Y., Zhang, Y., Zhang, M., Xu, L., Zheng, L., He, W., Yu, K., Li, T., et al. (2023). Disrupting the phase separation of KAT8-IRF1 diminishes PD-L1 expression and promotes antitumor immunity. Nat. Cancer *4*, 382–400. https://doi.org/10.1038/s43018-023-00522-1.
24. He, S., Luo, S., Cai, B., Chen, J., Zhang, Y., Zhao, F., Liu, Q., Liu, T., Wang, W., Peng, T., et al. (2025). Divergent roles of PKM2 in regulating PD-L1 and PD-L2 expression and their implications in human and mouse cancer models. Acta Biochim. Biophys. Sin. *57*, 1696–1708. https://doi.org/10.3724/abbs.2025019.
25. Komatsu, S., Ichikawa, D., Takeshita, H., Tsujiura, M., Morimura, R., Nagata, H., Kosuga, T., Iitaka, D., Konishi, H., Shiozaki, A., et al. (2011). Circulating microRNAs in plasma of patients with oesophageal squamous cell carcinoma. Br. J. Cancer *105*, 104–111. https://doi.org/10.1038/bjc.2011.198.
26. Morimura, R., Komatsu, S., Ichikawa, D., Takeshita, H., Tsujiura, M., Nagata, H., Konishi, H., Shiozaki, A., Ikoma, H., Okamoto, K., et al. (2011). Novel diagnostic value of circulating miR-18a in plasma of patients with pancreatic cancer. Br. J. Cancer *105*, 1733–1740. https://doi.org/10.1038/bjc.2011.453.
27. Kawaguchi, T., Komatsu, S., Ichikawa, D., Morimura, R., Tsujiura, M., Konishi, H., Takeshita, H., Nagata, H., Arita, T., Hirajima, S., et al. (2013). Clinical impact of circulating miR-221 in plasma of patients with pancreatic cancer. Br. J. Cancer *108*, 361–369. https://doi.org/10.1038/bjc.2012.546.
28. Hirajima, S., Komatsu, S., Ichikawa, D., Takeshita, H., Konishi, H., Shiozaki, A., Morimura, R., Tsujiura, M., Nagata, H., Kawaguchi, T., et al. (2013). Clinical impact of circulating miR-18a in plasma of patients with oesophageal squamous cell carcinoma. Br. J. Cancer *108*, 1822–1829. https://doi.org/10.1038/bjc.2013.148.
29. Arakawa, H., Komatsu, S., Kishimoto, T., Kamiya, H., Ishida, R., Takashima, Y., Nishibeppu, K., Kiuchi, J., Imamura, T., Ohashi, T., et al. (2025). Low levels of tumor suppressor miR-3619 in plasma contribute to malignant outcomes and a target for nucleic acid therapy in esophageal cancer. Sci. Rep. *15*, 27358. https://doi.org/10.1038/s41598-025-12246-6.

OMTN, Volume *37*

Supplemental information

Low circulating miR-5193 promotes PD-L1-mediated immune evasion and relates to immune checkpoint inhibitor response in esophageal cancer

Ryo Ishida, Shuhei Komatsu, Hajime Kamiya, Takuma Ohashi, Taisuke Imamura, Jun Kiuchi, Keiji Nishibeppu, Yusuke Takashima, Hiroshi Arakawa, Masateru Yamauchi, Satoshi Hamada, Hiroyuki Kanazawa, Hiroki Shimizu, Tomohiro Arita, Toshiyuki Kosuga, Hirotaka Konishi, Hitoshi Fujiwara, Tomoko Iehara, Hitoshi Tsuda, and Atsushi Shiozaki

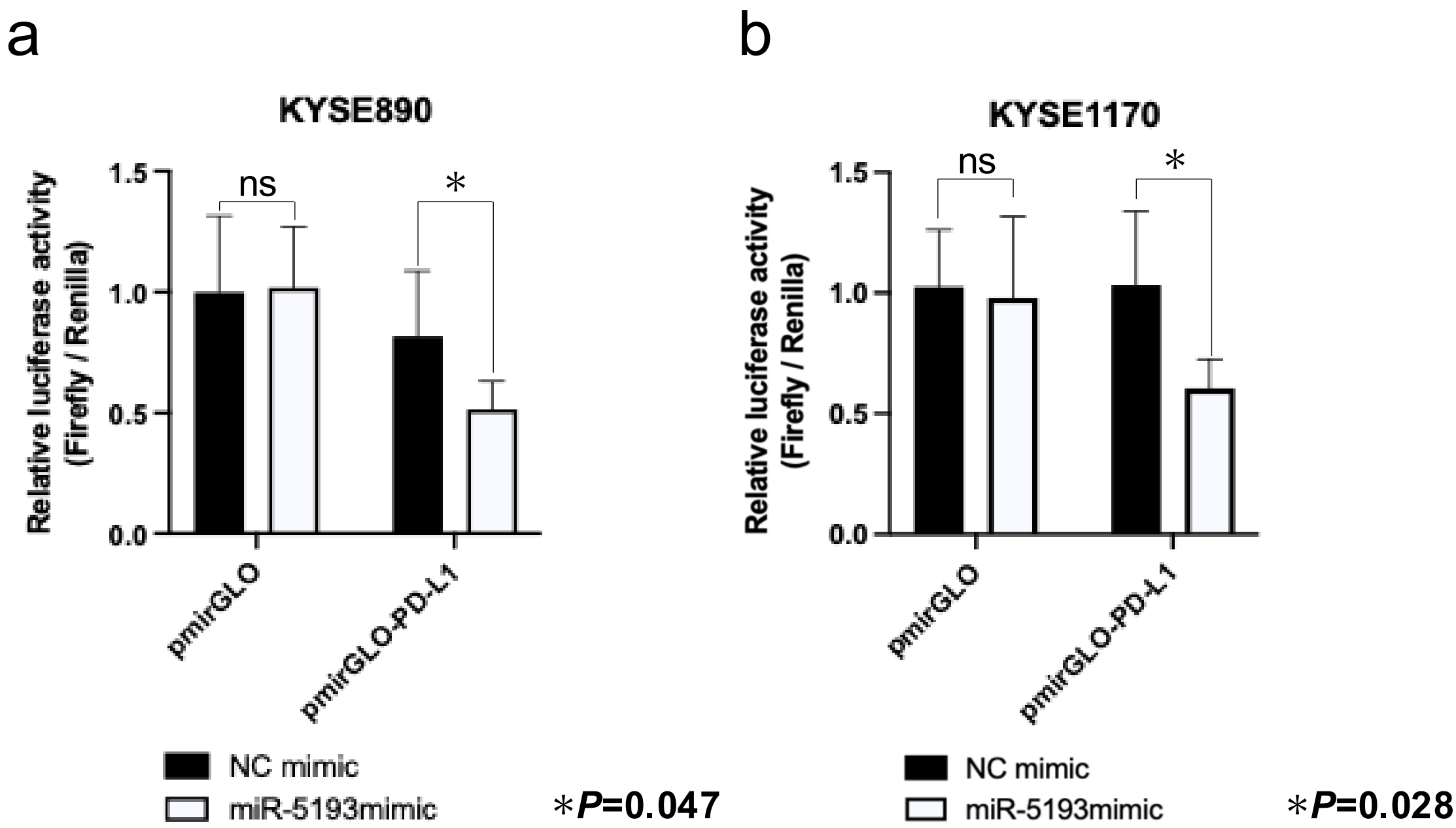

Figure S1

KYSE890 and KYSE1170 cells were co-transfected with an empty pmirGLO vector or a pmirGLO vector containing the PD-L1/CD274 3′ untranslated region (3′UTR), together with miR-5193 mimic or negative-control mimic. Luciferase activity was measured 48 h after transfection. Firefly luciferase activity was normalized to Renilla luciferase activity, and the relative luciferase activity of the negative-control mimic was set to 1 for each reporter construct. miR-5193 significantly reduced the luciferase activity of the PD-L1/CD274 3′UTR reporter.

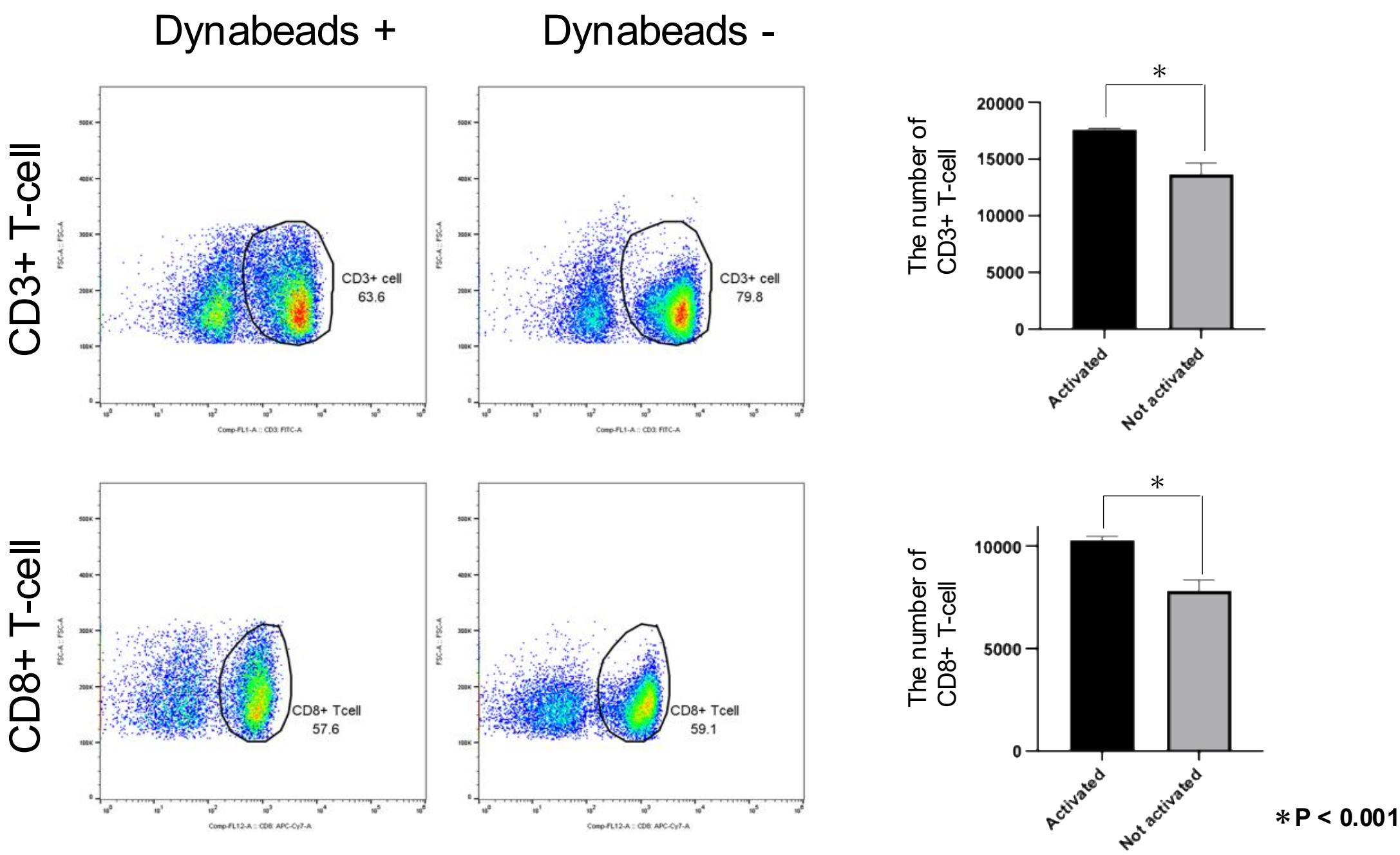

Figure S2

Peripheral blood mononuclear cell-derived T cells were stimulated with anti-CD3/CD28 Dynabeads, and $CD3^+$ and $CD8^+$ T-cell populations were evaluated by flow cytometry before and after stimulation. Anti-CD3/CD28 stimulation increased the absolute numbers of $CD3^+$ and $CD8^+$ T cells, whereas the proportion of $CD8^+$ cells among $CD3^+$ T cells was not markedly altered. These data were used as a quality-control assessment of the activated T-cell population used in the co-culture assay.

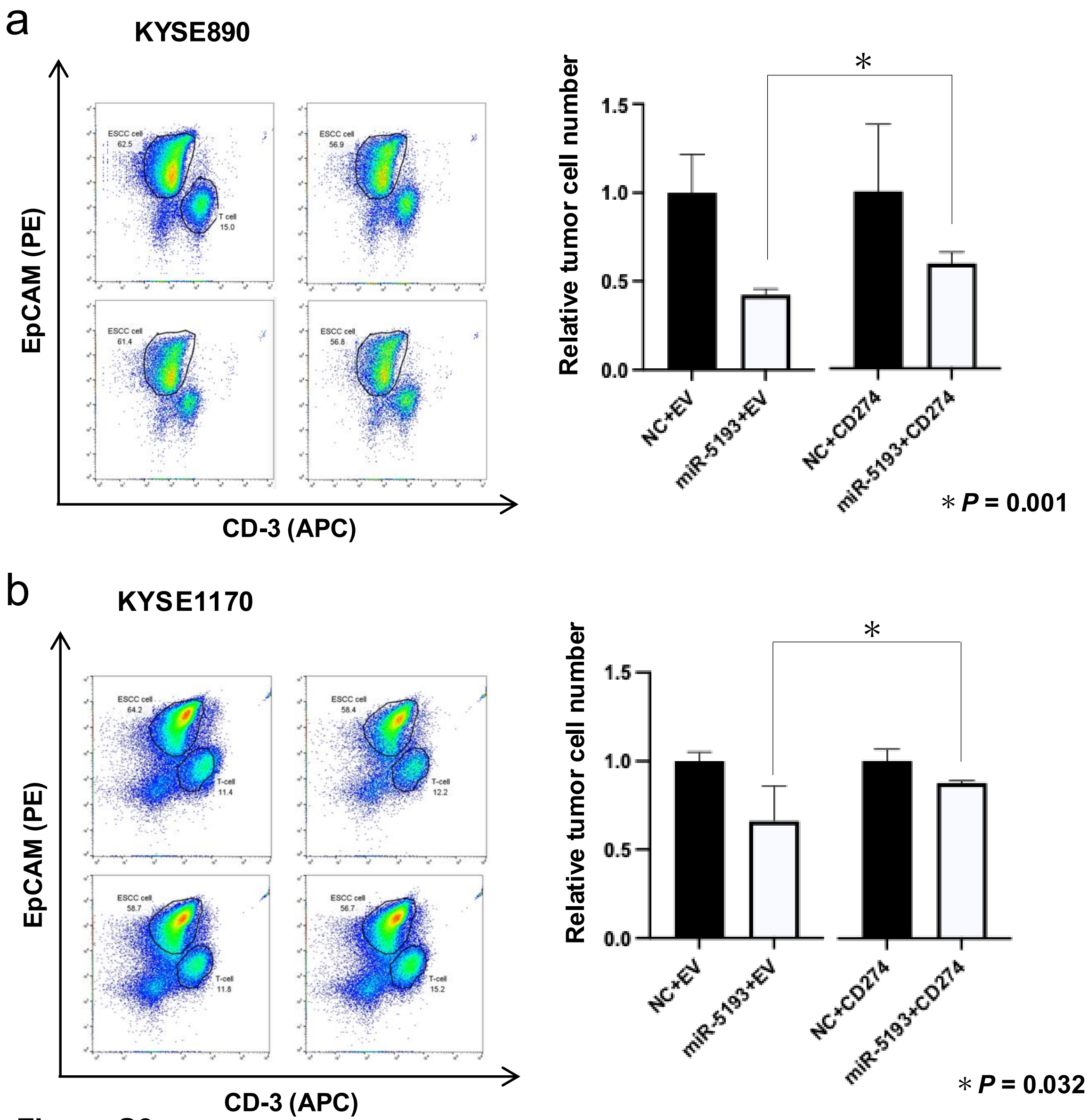

Figure S3

KYSE890 and KYSE1170 cells were transfected with miR-5193 mimic or negative-control mimic, followed by transient transfection with a human CD274/PD-L1 open reading frame expression vector or the corresponding empty vector. The cells were then co-cultured with activated $CD3^+$ T cells. Viable tumor cells were quantified as epithelial cell adhesion molecule-positive, CD3-negative cells by flow cytometry using counting beads. CD274/PD-L1 re-expression partially attenuated the miR-5193 mimic-induced reduction in viable tumor cells, suggesting that miR-5193 enhances T-cell–mediated antitumor activity at least in part through PD-L1 suppression.

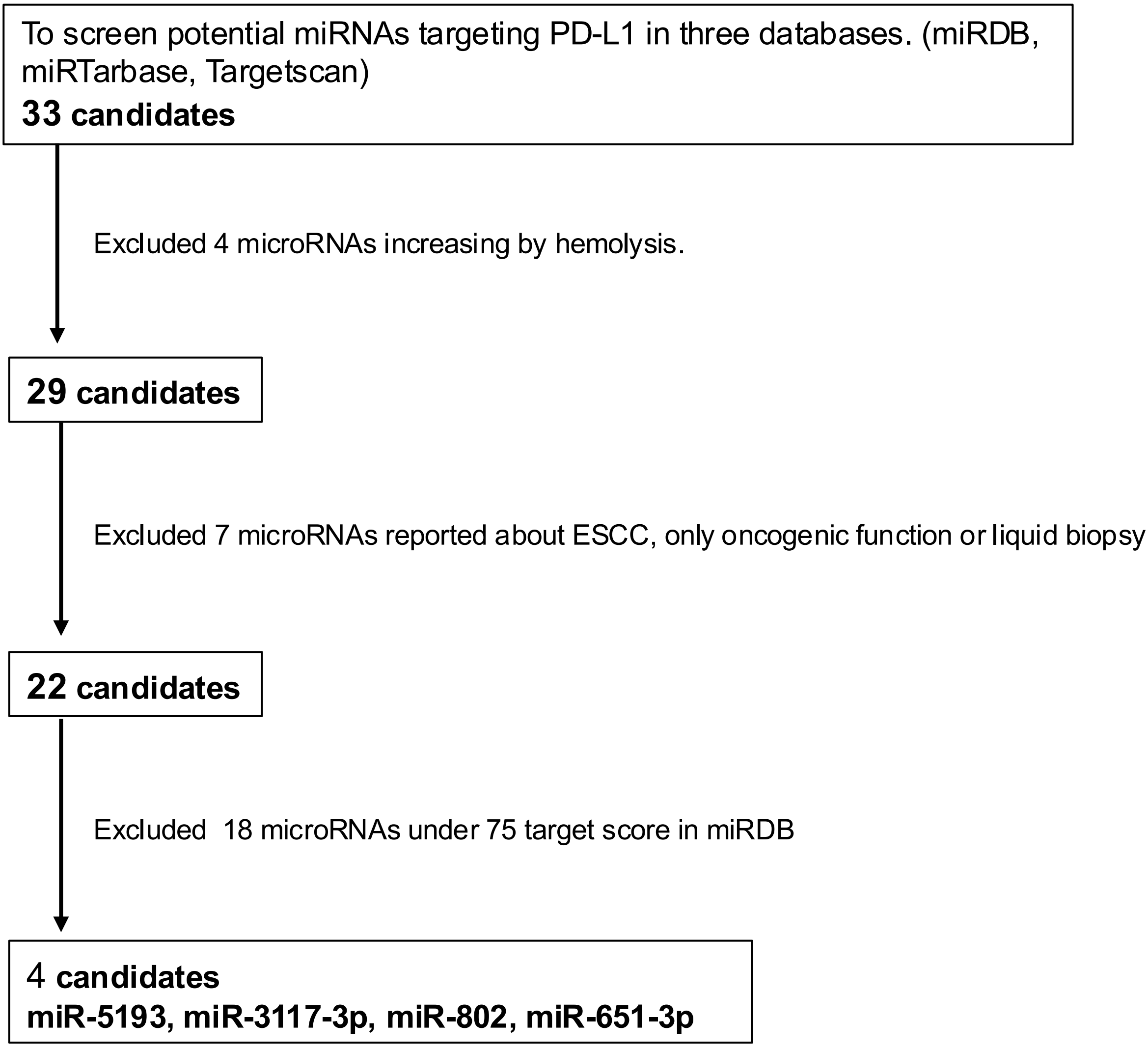

Figure S4

Candidate miRNAs predicted to target PD-L1/CD274 were identified using public miRNA target prediction/validation databases, including miRDB, miRTarBase, and TargetScan. miRNAs supported by multiple databases were prioritized, and candidates previously reported to be hemolysis-related, mainly oncogenic, or already described in upper gastrointestinal cancers were excluded. Four candidate miRNAs, miR-5193, miR-3117-3p, miR-802, and miR-651-3p, were selected for experimental evaluation.
